# A Mini-Atlas of Gene Expression for the Domestic Goat (*Capra hircus*)

**DOI:** 10.3389/fgene.2019.01080

**Published:** 2019-11-04

**Authors:** Charity Muriuki, Stephen J. Bush, Mazdak Salavati, Mary E.B. McCulloch, Zofia M. Lisowski, Morris Agaba, Appolinaire Djikeng, David A. Hume, Emily L. Clark

**Affiliations:** ^1^The Roslin Institute and Royal (Dick) School of Veterinary Studies, University of Edinburgh, Edinburgh, United Kingdom; ^2^Centre for Tropical Livestock Genetics and Health (CTLGH), Edinburgh, United Kingdom; ^3^Nuffield Department of Clinical Medicine, John Radcliffe Hospital, University of Oxford, Oxford, United Kingdom; ^4^Biosciences Eastern and Central Africa - International Livestock Research Institute (BecA - ILRI) Hub, Nairobi, Kenya; ^5^Mater Research Institute-University of Queensland, Woolloongabba, QLD, Australia

**Keywords:** goat, transcriptomics, RNA-Seq, gene expression, FAANG, allele-specific expression, immunity, comparative transcriptomics

## Abstract

Goats (*Capra hircus*) are an economically important livestock species providing meat and milk across the globe. They are of particular importance in tropical agri-systems contributing to sustainable agriculture, alleviation of poverty, social cohesion, and utilisation of marginal grazing. There are excellent genetic and genomic resources available for goats, including a highly contiguous reference genome (ARS1). However, gene expression information is limited in comparison to other ruminants. To support functional annotation of the genome and comparative transcriptomics, we created a mini-atlas of gene expression for the domestic goat. RNA-Seq analysis of 17 transcriptionally rich tissues and 3 cell-types detected the majority (90%) of predicted protein-coding transcripts and assigned informative gene names to more than 1000 previously unannotated protein-coding genes in the current reference genome for goat (ARS1). Using network-based cluster analysis, we grouped genes according to their expression patterns and assigned those groups of coexpressed genes to specific cell populations or pathways. We describe clusters of genes expressed in the gastro-intestinal tract and provide the expression profiles across tissues of a subset of genes associated with functional traits. Comparative analysis of the goat atlas with the larger sheep gene expression atlas dataset revealed transcriptional similarities between macrophage associated signatures in the sheep and goats sampled in this study. The goat transcriptomic resource complements the large gene expression dataset we have generated for sheep and contributes to the available genomic resources for interpretation of the relationship between genotype and phenotype in small ruminants.

## Introduction

Goats (*Capra hircus*) are an important source of meat and milk globally. They are an essential part of sustainable agriculture in low- and middle-income countries, representing a key route out of poverty particularly for women. Genomics-enabled breeding programmes for goats are currently implemented in the UK and France with breeding objectives including functional traits such as reproductive performance and disease resistance (Larroque et al., 2016; Pulina et al., 2018). The International Goat Genomics Consortium (IGGC) (http://www.goatgenome.org) has provided extensive genetic tools and resources for goats including a 52K SNP chip (Tosser-Klopp et al., 2014), a functional SNP panel for parentage assessment and breed assignment (Talenti et al., 2018) and large-scale genotyping datasets characterising global genetic diversity (Stella et al., 2018). In 2017, a highly contiguous reference genome for goat (ARS1) was released (Bickhart et al., 2017; Worley, 2017). Advances in genome sequencing technology, particularly the development of long-read and single-molecule sequencing, meant that the ARS1 assembly was a considerable improvement in quality and contiguity from the previous whole genome shotgun assembly (CHIR_2.0) (Dong et al., 2013). In 2018, the ARS1 assembly was released on the Ensembl genome portal (Zerbino et al., 2018) (https://www.ensembl.org/Capra_hircus/Info/Index) greatly facilitating the utility of the new assembly and providing a robust set of gene models for goat.

RNA-Sequencing (RNA-Seq) has transformed the analysis of gene expression from the single-gene to the whole genome allowing visualisation of the entire transcriptome and defining how we view the transcriptional control of complex traits in livestock [reviewed in (Wickramasinghe et al., 2014)]. Using RNA-Seq, we generated a large-scale high-resolution atlas of gene expression for sheep (Clark et al., 2017). This dataset included RNA-Seq libraries from all organ systems and multiple developmental stages, providing a model transcriptome for ruminants. Analysis of the sheep gene expression atlas dataset indicated we could capture approximately 85% of the transcriptome by sampling twenty 'core' tissues and cell types (Clark et al., 2017). Given the close relationship between sheep and goats, there seemed little purpose in replicating a resource on the same scale. Our aim with the goat mini-atlas project, which we present here, was to produce a smaller, cost-effective, atlas of gene expression for the domestic goat based on transcriptionally rich tissues from all the major organ systems.

In the goat genome, there are still many predicted protein-coding and noncoding genes for which the gene model is either incorrect or incomplete, or where there is no informative functional annotation. For example, in the current goat reference genome, ARS1 (Ensembl release 97), 33% of the protein-coding genes are identified only with an Ensembl placeholder ID. Many of these unannotated genes are likely to have important functions. Using RNA-Seq data, we can annotate them and assign function (Krupp et al., 2012). With datasets of a sufficient size, genes form coexpression clusters, which can either be ubiquitous, associated with a cellular process or be cell-/tissue specific. This information can then be used to associate a function with genes coexpressed in the same cluster, a method of functional annotation known as the “guilt by association principle” (Oliver, 2000). Using this principle with the sheep gene expression atlas dataset, we were able to annotate thousands of previously unannotated transcripts in the sheep genome (Clark et al., 2017). By applying this rationale to the goat mini-atlas dataset we were able to do the same for the goat genome.

The goat mini-atlas dataset that we present here was used by Ensembl to create the initial gene build for ARS1 (Ensembl release 92). A high-quality functional annotation of existing reference genomes can help considerably in our understanding of the transcriptional control of functional traits to improve the genetic and genomic resources available, inform genomics enabled breeding programmes, and contribute to further improvements in productivity. The entire dataset is available in a number of formats to support the livestock genomics research community and represents an important contribution to the Functional Annotation of Animal Genomes (FAANG) project (Andersson et al., 2015; FAANG, 2017; Harrison et al., 2018).

This study is the first global analysis of gene expression in goats. Using the goat mini-atlas dataset, we describe large clusters of genes associated with the gastrointestinal tract and macrophages. Species specific differences in response to disease, or other traits, are likely to be reflected in gene expression profiles. Sheep and goats are both small ruminant mammals and are similar in their physiology. They also share susceptibility to a wide range of viral, bacterial, parasitic, and prion pathogens, including multiple potential zoonoses (Sherman, 2011), but there have been few comparisons of relative susceptibility or pathology between the species to the same pathogen nor the nature of innate immunity. To reveal transcriptional similarities and differences between sheep and goats, we have performed a comparative analysis of gene expression by comparing the goat mini-atlas dataset with a comparable subset of data from the sheep gene expression atlas (Clark et al., 2017). We also use the goat mini-atlas dataset to examine the expression of candidate genes associated with functional traits in goats and link these with allele-specific expression (ASE) profiles across tissues, using a robust methodology for ASE profiling (Salavati et al., 2019). The goat mini-atlas dataset and the analysis we present here provide a foundation for identifying the regulatory and expressed elements of the genome that are driving functional traits in goats.

## Methods

### Animals

Tissue and cell samples were collected from six male and one female neonatal crossbred dairy goats at six days old. The experimental design was based on sample availability at the time of the study. The goats were sourced from one farm and samples were collected at a local abattoir within 1 h of euthanasia.

### Tissue Collection

The tissue samples were excised postmortem within 1 h of death, cut into 0.5cm diameter segments, and transferred into RNAlater (Thermo Fisher Scientific, Waltham, USA) and stored at 4°C for short-term storage. Within one week, the tissue samples were removed from the RNAlater, transferred to 1.5ml screw cap cryovials, and stored at -80°C until RNA isolation. Alveolar macrophages (AMs) were isolated from two male goats by broncho-alveolar lavage of the excised lungs using the method described for sheep in (Clark et al., 2017), except using 20% heat-inactivated goat serum (G6767, Sigma Aldrich), and stored in TRIzol (15596018; Thermo Fisher Scientific) at -80°C for RNA extraction. Similarly, bone marrow cells (BMCs) were isolated from 10 ribs from 3 male goats and frozen down for subsequent differentiation and stimulation with lipopolysaccharide (LPS) using the method described in (Clark et al., 2017; Young et al., 2018). Bone marrow derived macrophages (BMDMs) were obtained by culturing BMCs for 10 days in complete medium: RPMI 1640, Glutamax supplement (35050–61; Invitrogen), 20% heat inactivated goat serum (G6767; Sigma Aldrich), penicillin/streptomycin (15140, Invitrogen), and in the presence of recombinant human CSF-1 (rhCSF-1: 10^4^ U/ml; a gift of Chiron, Emeryville, CA) on T75 polystyrene tissue culture treated plates (156499; Thermo Fisher Scientific) at a density of 2.0x10^6^cells/ml. On day 11, BMDMs were transferred to 6-well cell culture treated multidishes (140675; Thermo Fisher Scientific). The following day, they were stimulated with LPS from *Salmonella enterica* serotype minnesota Re 595 (L9764; Sigma-Aldrich) at a final concentration of 100 ng/ml, then transferred into TRIzol (15596018; Thermo Fisher Scientific) at 0, 7h post LPS treatment, and stored at -80°C for RNA extraction.

Details of all the samples collected are included in **Table 1**.

**Table 1:** Details of samples included in the goat mini-atlas.

Tissue/cell type	Organ system	No. of replicates	Sex
**Adrenal gland**	Endocrine	4	male
**Alveolar macrophage**	Immune	2	male
**BMDM - LPS (0 h)**	Immune	3	male
**BMDM + LPS (7 h)**	Immune	3	male
**Cerebellum**	Nervous system	2	male
**Colon large**	GI tract	4	male
**Fallopian tube**	Reproductive system	1	female
**Frontal lobe cortex**	Nervous system	2	male
**Ileum and Peyer’s patches**	GI tract	2	male
**Kidney cortex**	Endocrine	4	male
**Liver**	Endocrine	4	male
**Ovary**	Reproductive system	1	female
**Rumen**	Gastrointestinal tract	2	male
**Skeletal muscle - longissimus dorsi**	Musculo-skeletal	3	male
**Skin**	Integumentary	4	male
**Spleen**	Immune	3	male
**Testes**	Reproductive system	4	male
**Thymus**	Immune	4	male
**Uterine horn**	Reproductive system	1	female
**Uterus**	Reproductive system	1	female

### RNA Extraction

RNA was extracted from tissues and cells as described in (Clark et al., 2017). For each RNA extraction from tissues, approximately 60mg of tissue was processed. Tissue samples were homogenised on a Precellys Tissue Homogeniser (Bertin Instruments; Montigny-le-Bretonneux, France) at 5000 rpm for 20 s with CK14 (432–3751; VWR, Radnor, USA) tissue homogenising ceramic beads in 1ml of TRIzol (15596018; Thermo Fisher Scientific). Cell samples were collected atthe point of isolation into TRIzol (15596018; Thermo Fisher Scientific), stored at -80°C, thawed, and then mixed by pipetting to homogenise. To allow sufficient time for complete dissociation of the nucleoprotein complex, homogenised (cell/tissue) samples were incubated at room temperature for 5 min. After 5 min, 200µl BCP (1-bromo-3-chloropropane) (B9673; Sigma Aldrich) was added and the sample was shaken vigorously for 15 s and incubated at room temperature for a further 3 min. The homogenised sample was then centrifuged for 15 min at 12,000 x *g*, at 4°C for 3 min, to separate the upper clear aqueous layer. This clear upper layer was then column purified to remove DNA and trace phenol using a RNeasy Mini Kit (74106; Qiagen Hilden, Germany) following the manufacturer’s instructions (RNeasy Mini Kit Protocol: Purification of Total RNA from Animal Tissues, from step 5 onwards). An on-column DNase treatment was performed using the Qiagen RNase-Free DNase Set (79254; Qiagen Hilden, Germany). The sample was eluted in 30ul of RNase free water and stored at -80°C prior to QC and library preparation. To ensure RNA integrity (RIN^e^) was of RIN^e^ > 7 samples were run on an Agilent 2200 TapeStation System (Agilent Genomics, Santa Clara, USA). RIN^e^ and other quality control metrics for the RNA samples are included in **Supplementary Table S1**.

### RNA-Sequencing

RNA-Seq libraries were sequenced on the Illumina HiSeq 4000 sequencing platform (Illumina, San Diego, USA) and generated by Edinburgh Genomics (Edinburgh Genomics, Edinburgh, UK). Strand-specific paired-end reads with a fragment length of 75bp were generated for each sample using the standard Illumina TruSeq mRNA library preparation protocol (poly-A selected) (Ilumina; Part: 15031047 Revision E). Libraries were sequenced at a depth of either >30 million reads per sample for the tissues and AMs, or >50 million reads per sample for the BMDMs.

### Data Processing

The RNA-Seq data processing methodology and pipelines are described in detail in (Clark et al., 2017). Briefly, for each tissue, a set of expression estimates, as transcripts per million (TPM), were obtained. These estimates were obtained using the alignment-free (technically, “pseudo-aligning”) transcript quantification tool Kallisto (Bray et al., 2016), the accuracy of which is dependent on a high quality reference transcriptome (index). We used a “two-pass” approach to generate this index in order to ensure we used an accurate set of gene expression estimates.

To generate the index, we initially ran Kallisto on all samples using as its index the ARS1 reference transcriptome available from Ensembl (ftp://ftp.ensembl.org/pub/release-95/fasta/capra_hircus/cdna/Capra_hircus.ARS1.cdna.all.fa.gz). The resulting data was then parsed to revise this index. This was for two reasons: i) so that we included in the second index, those transcripts that were missing but should have been present (i.e. due to incompleteness in the reference annotation), and ii) to remove transcripts that were present but should not have been (i.e., where a spurious model was present in the reference annotation). For i), we obtained the subset of reads that Kallisto could not (pseudo)align, assembled those *de novo* into putative transcripts, then retained each transcript only if it could be robustly annotated. We considered annotation robust when a transcript encoded a protein similar to one of known function and had coding potential. For ii), we identified those transcripts in the reference transcriptome for which no evidence of expression could be found in any of the samples from the goat mini-atlas and discarded them. This revised index was used for a second “pass” with Kallisto to generate expression level estimates with higher-confidence.

We complemented the Kallisto alignment-free method with a reference-guided alignment-based approach to RNA-Seq processing, using the HISAT aligner (Kim et al., 2015) and StringTie assembler (Pertea et al., 2015). This approach was highly accurate when mapping to the (ARS1) annotation on NCBI (ftp://ftp.ncbi.nlm.nih.gov/genomes/all/GCF/001/704/415/GCF_001704415.1_ARS1/GCF_001704415.1_ARS1_rna.fna.gz), precisely reconstructing almost all exon (96%) and transcript (76%) models (**Supplementary Table S2**). We used the HISAT/StringTie output to validate the set of transcripts used to generate the Kallisto index. HISAT/StringTie, unlike Kallisto and other alignment-free methods, can be used to identify novel transcript models, particularly for ncRNAs, which we have described separately in (Bush et al., 2018b). Details of all novel transcript models detected are included in **Supplementary Table S3**.

### Data Validation

To identify any spurious samples which could have been generated during sample collection, RNA extraction, or library preparation, we generated a sample-to-sample correlation of the gene expression estimates from Kallisto, in Graphia Professional (Kajeka Ltd, Edinburgh, UK).

### Network Cluster Analysis

Network cluster analysis of the goat gene mini-atlas dataset was performed using Graphia Professional (Kajeka Ltd, Edinburgh, UK) (Livigni et al., 2018). Briefly, by calculating a Pearson correlation matrix for both gene-to-gene and sample-to-sample comparisons, and filtering to remove relationships where *r* < 0.83, we were able to determine similarities between individual gene expression profiles. A network graph was constructed by connecting the nodes (transcripts) with edges (where the correlation exceeded the threshold value). Network graphs were interpreted by applying a Markov Cluster algorithm (MCL) at an inflation value/cluster granularity of 2.2 (Freeman et al., 2007). The granularity of the network graph was manually curated in order to reach a biologically relevant number of interaction nodes and cluster numbers. This approach was iteratively applied to several correlation coefficient thresholds for comparison prior to clustering, as previously described in Freeman et al., 2007, Clark et al., 2017. A suitable correlation threshold of 0.83 was chosen and the local structure of the graph was then examined visually. Transcripts with related functions clustered together forming sets of tightly interlinked nodes. The principle of “guilt by association” was then applied, to infer the function of unannotated genes from genes within the same cluster (Oliver, 2000). Clusters 1 to 30 were assigned a functional “class” based on whether transcripts within a cluster shared a similar biological function according to GO term enrichment using the Bioconductor package “topGO” (Alexa and Rahnenfuhrer, 2010).

### Comparative Analysis of Gene Expression in Macrophages in Sheep and Goats

To compare transcriptional differences in the immune response between the two species, we focused our analysis on the macrophage populations (AMs and BMDMs). For this analysis, we used a subset of data from our sheep gene expression atlas for AMs and BMDMs (+/- LPS) from three male sheep (Clark et al., 2017) (**Supplementary Dataset S1**).

For AMs, we compared the gene level expression estimates from the two male goats and the three male sheep using edgeR v3.20.9 (Robinson et al., 2010). Only genes with the same gene name in both species, expressed at a raw read count of more than 10, FDR < 10%, an FDR adjusted p-value of <0.05, and Log2FC of > = 2, in both goat and sheep, were included in the analysis.

Differential expression analysis using edgeR (Robinson et al., 2010) was also performed for sheep and goat BMDMs (+/-) LPS separately, using the filtration criteria described above for AMs, to compile a list of genes for each species that were up or down regulated in response to LPS. These lists were then compared using the R package dplyr (Wickham et al., 2018) with system query language syntax. Each list was merged based on GENE_ID using the *inner_join* function to only return the observations that overlapped between goat and sheep (i.e., genes which had corresponding annotations in both species).

A dissimilarity index (Dis_Index) was then calculated by taking the absolute difference (ABS) of the Log2 fold change (Log2FC) between sheep and goat using the formula:

$$\text{ABS}({\text{Log2FC}}^{\text{Sheep}}{\text{-Log2FC}}^{\text{Goat}})$$

A high Dis_Index indicated that a gene was differently regulated in goat and sheep.

### Allele-Specific Expression

To measure allele-specific expression (ASE), across tissues and cell-types from the goat mini-atlas, we used the method described in (Salavati et al., 2019). Briefly, BAM files from the RNA-Seq data were mapped to the ARS1 top level DNA fasta track from Ensembl v96, using HISAT2 as described in (Clark et al., 2017). Any reference mapping bias was removed using WASP v0.3.1 (van de Geijn et al., 2015) and the resultant BAM files processed using the Genome Analysis Tool Kit (GATK) to produce individual VCF files. The ASEreadCounter tool in GATK v3.8 was used to obtain raw counts of the allelic expression profile in the dataset. These raw counts were then tested for imbalance (using a modified negative-beta bionomial test at gene level) at all heterozygote loci (i.e., ASE = Counts _RefAllele_/(Counts _RefAllele_+ Counts _AltAllele_) within the boundaries of the gene using the R package GeneiASE (Edsgärd et al., 2016).

## Results and Discussion

### Scope of the Goat Mini-Atlas Dataset, Sequencing Depth, and Coverage

The goat mini-atlas dataset includes 54 mRNA-Seq (poly-A selected) 75bp paired-end libraries. Details of the libraries generated including the age and sex of the animals, the tissues and cell types sampled, and the number of biological replicates per sample are summarised in **Table 1**. Gene level expression estimates, for the goat mini-atlas, are provided as unaveraged (**Supplementary Dataset S2**) and averaged across biological replicates (**Supplementary Dataset S3**) files.

Approximately, 8.7x10^8^ paired end sequence reads were generated in total. Following data processing with Kallisto (Bray et al., 2016), a total of 18,528 unique protein coding genes had detectable expression (TPM > 1), representing 90% of the reference transcriptome (Bickhart et al., 2017). From the set of 17 tissues and 3 cell types we sampled, we were able to detect approximately 90% of protein coding genes providing proof of concept that the mini-atlas approach is useful for global analysis of transcription. The average percentage of transcripts detected per tissue or cell type was 66%, ranging from 54% in alveolar macrophages, which had the lowest to 72% in testes, which had the highest. The percentage of protein coding genes detected per tissue is included in **Table 2**. Although we included uterine horn as well as uterus and both stimulated and unstimulated BMDMs, our analysis suggests that including only one tissue/cell of a similar type would be the most economical approach to generating a mini-atlas of gene expression for functional annotation.

**Table 2:** The percentage of protein coding genes detected per tissue in the goat mini-atlas dataset.

Tissue	Average no. of protein-coding genes expressed (TPM > 1) in this tissue	% of protein-coding genes expressed (TPM > 1) in this tissue
**Adrenal gland**	14585	68.34
**Alveolar macrophage**	11533	54.04
**BMDM - LPS (0 h)**	13253	62.1
**BMDM + LPS (7 h)**	13042	61.11
**Cerebellum**	14959	70.09
**Colon large**	14736	69.04
**Fallopian tube**	14390	67.42
**Frontal lobe cortex**	14757	69.14
**Ileum and Peyer’s patches**	15268	71.54
**Kidney cortex**	15223	71.33
**Liver**	13497	63.24
**Ovary**	14251	66.77
**Rumen**	13642	63.92
**Skeletal muscle - longissimus dorsi**	12276	57.52
**Skin**	14892	69.77
**Spleen**	14659	68.68
**Testes**	15359	71.96
**Thymus**	14484	67.86
**Uterine horn**	14298	66.99
**Uterus**	14298	66.99

Approximately, 2,815 (13%) of the total 21,343 protein coding genes in the goat reference transcriptome had no detectable expression in the goat mini-atlas dataset. These transcripts are likely to be either tissue specific to tissues and cell-types that were not sampled here (including lung, heart, pancreas, and various endocrine organs), rare, or not detected at the depth of coverage used. The large majority of these transcripts were detected in the much larger sheep atlas, and their likely expression profile can be inferred from the sheep. In addition, for the goat mini-atlas unlike the sheep gene expression atlas, we only included neonatal animals so transcripts that were highly developmental stage-specific in their expression pattern would also not be detected. A list of all undetected genes is included in **Supplementary Table S4** and undetected transcripts in **Supplementary Table S5**.

### Gene Annotation

The proportion of transcripts per biotype (lncRNA, protein coding, pseudogene, etc), with detectable expression (TPM >1) in the goat mini-atlas relative to the ARS1 reference transcriptome, on Ensembl is summarised at the gene level in **Supplementary Table S6** and at the transcript level in **Supplementary Table S7**. Of the 21,343 protein coding genes in the ARS1 reference transcriptome, 7036 (33%) had no informative gene name. Whilst the Ensembl annotation will often identify homologues of a goat gene model, the automated annotation genebuild pipeline used to assign gene names and symbols is conservative. Using the annotation pipeline we described in (Clark et al., 2017), we were able to use the goat mini-atlas dataset to assign an informative gene name to 1114 previously unannotated protein coding genes in ARS1. These genes were annotated by reference to the NCBI nonredundant (nr) peptide database v94 (Pruitt et al., 2007). A shortlist containing a conservative set of gene annotations to HGNC (HUGO Gene Nomenclature Committee) gene symbols is included in **Supplementary Table S8**. **Supplementary Table S9** contains the full list of genes annotated using the goat mini-atlas dataset and our annotation pipeline. Many unannotated genes can be associated with a gene description, but not necessarily an HGNC symbol; these are also listed in **Supplementary Table S10**.

### Network Cluster Analysis

**Figure 1:**
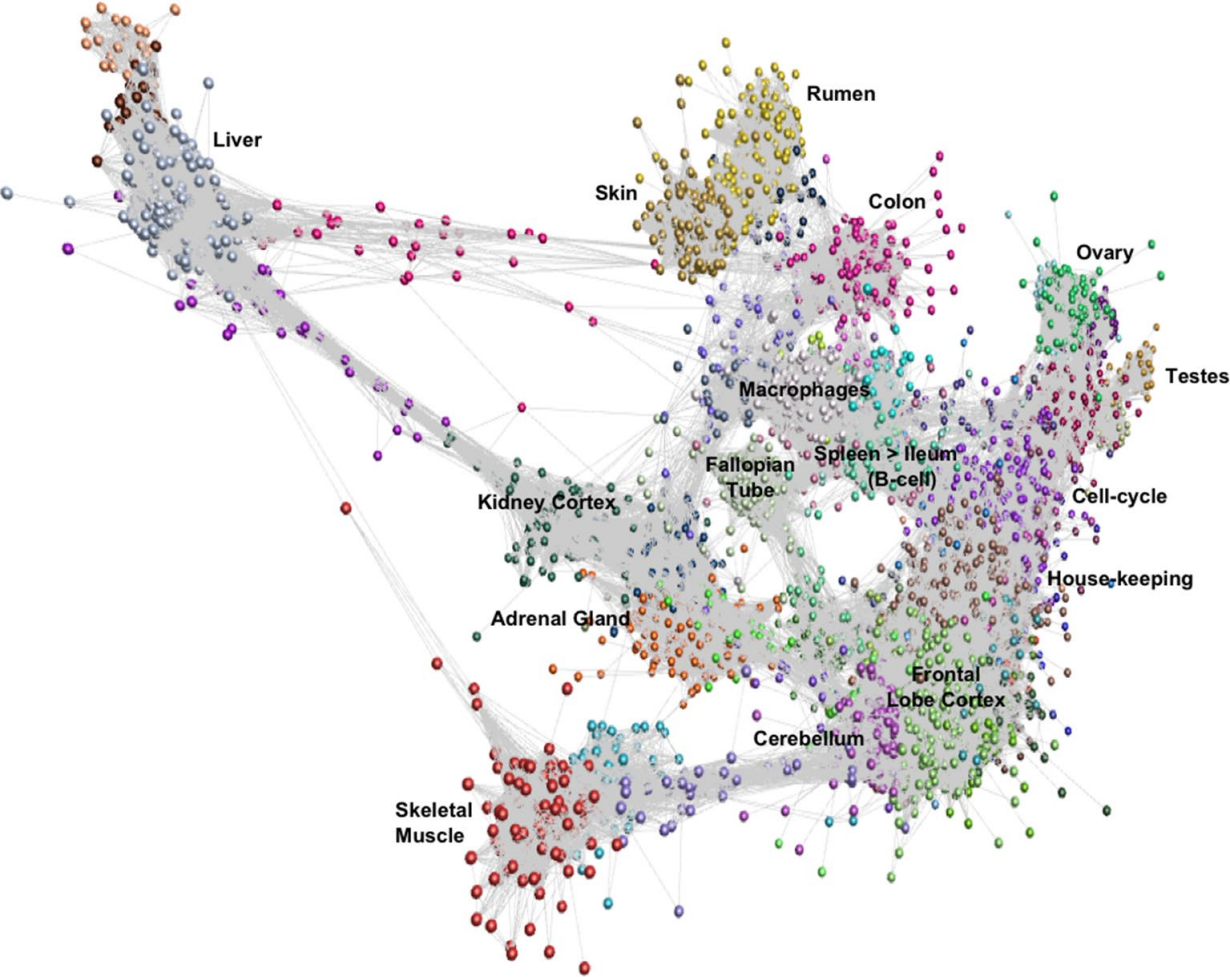
Gene-to-gene network graph of the goat mini-atlas dataset. Each node represents a gene and each edge represents correlations between individual measurements above the set threshold. The graph comprised 16,172 nodes (genes) and 1,574,259 edges (Pearson correlations ≥ 0.83), Markov Cluster algorithm (MCL) inflation = 2.2, and Pearson Product Correlation Co-efficient = 0.83. (> indicates decreasing expression profile).

Network cluster analysis of the goat gene expression atlas was performed using Graphia Professional (Kajeka Ltd, Edinburgh UK), a network visualisation tool (Livigni et al., 2018). The goat mini-atlas unaveraged TPM estimates (**Supplementary Dataset S2**) were used for network cluster analysis. We first generated a sample-to-sample graph (r = 0.75, MCL = 2.2) **Supplementary Figure S1**, which verified that the correlation between biological replicates was high and that none of the samples were spurious. We then generated a gene-to-gene network graph (**Figure 1**), with a Pearson correlation coefficient of r = 0.83, that comprised 16,172 nodes (genes) connected by 1,574,259 edges. The choice of Pearson correlation threshold is optimised within the Graphia program to maximise the number of nodes (genes) included whilst minimising the number of edges (Freeman et al., 2007). By applying the MCL (Markov Clustering) algorithm at an inflation value (which determines cluster granularity) of 2.2, the gene network graph separated into 75 distinct coexpression clusters, with the largest cluster (cluster 1) comprising of 1795 genes. Genes found in the top 30 largest clusters are listed in **Supplementary Table S11**. Clusters 1 to 20 (numbered in order of size, largest to smallest) were annotated manually and assigned a functional “class” (**Table 3**). These functional classes were assigned based on GO term enrichment (Alexa and Rahnenfuhrer, 2010) for molecular function and biological process (**Supplementary Table S12**). Assignment of functional class was further validated by visual inspection of expression pattern and comparison with functional groupings of genes observed in the sheep gene expression atlas (Clark et al., 2017).

**Table 3:** Annotation of the 20 largest network clusters in the goat mini-atlas dataset (> indicates decreasing expression profile).

Cluster ID	Number of genes	Profile description	Class	Enriched GO terms
**1**	1795	Cortex > cerebellum	Brain	cognition, neurotransmitter transport, synaptic transmission
**2**	1395	Thymus > Spleen > Ileum	Cell-Cycle	DNA-dependent DNA replication, DNA repair
**3**	795	General	House Keeping	mRNA processing, regulation of RNA splicing
**4**	505	Liver	Oxidative-Phosphorylation	oxidation-reduction process, fatty acid oxidation
**5**	494	General	House Keeping	RNA binding, nucleolus
**6**	481	Testes	Male Reproduction	male meiosis, spermatogenesis
**7**	449	Skin > Rumen	Epithelial	skin morphogenesis, keratinocyte differentiation
**8**	374	Fallopian Tube	Motile Cilia	motile cilium, ciliary basal body
**9**	351	Skeletal muscle	Muscle	muscle fibre development, motor activity
**10**	337	Spleen > Ileum	Immune	immune response, B-cell activation, cytokine activity
**11**	290	Macrophages	Immune	response to lipopolysaccharide, phagocytic vesicle
**12**	241	Colon Large	Gastrointestinal tract	microvillus, actin filament bundle
**13**	226	Rumen > Skin	Gastrointestinal/Epithelial	epidermis development, chloride channel activity
**14**	219	Adrenal Gland	Endocrine	oxidation-reduction process, sterol metabolic process
**15**	211	BMDMs	Fibroblasts	collagen binding, positive regulation of fibroblast proliferation
**16**	134	General	Ribosomal	ribosomal large subunit biogenesis, ribosome
**17**	133	Kidney Cortex	Mesoendonephric organogenesis	sodium ion homeostasis, skeletal system morphogenesis
**18**	119	Ovary	Oogenesis	growth factor activity, nucleosome disassembly
**19**	113	Ileum > Spleen > Thymus	Immune	B-cell proliferation, cytokine activity
**20**	108	Uterus, Uterine Horn	Organogenesis	tissue remodelling, bone morphogenesis

The largest of the clusters (Cluster 1) contained 1,795 genes that were almost exclusively expressed in the central nervous system (cortex, cerebellum) reflecting the high transcriptional activity and complexity in the brain. Significant GO terms for cluster 1 included cognition (p = 4.6x10^-17^) and synaptic transmission (p = 2.5x10^-30^). Other tissue-specific clusters, e.g., 4 (liver), 6 (testes), 7 (skin/rumen), 14 (adrenal), and 17 (kidney), were similarly enriched for genes associated with known tissue-specific functions. In each case, the likely function of unannotated protein-coding genes within these clusters could be inferred by association with genes of known function that share the same cell or tissue specific expression pattern. Cluster 9 showed a high level of tissue specificity and included genes associated with skeletal muscle function and development including *MSTN* which encodes a protein that negatively regulates skeletal muscle cell proliferation and differentiation (Wang et al., 2012). Several myosin light and heavy chain genes (e.g., *MYH1* and *MYL1*) and transcription factors that are specific to muscle including (*MYOG* and *MYOD1*) were also found in cluster 9. GO terms for muscle were enriched in cluster 9, e.g., muscle fiber development (p = 3.8x10^-13^) and structural constituent of muscle (p = 1.8x10^-11^). Genes expressed in muscle are of particular biological and commercial interest for livestock production and represent potential targets for gene editing (Yu et al., 2016). Cluster 8 was also highly tissue specific and included genes expressed in the fallopian tube with enriched GO terms for cilium movement (p = 1.4x10^-15^) and cilium organization (p = 2.3x10^-15^). A motile cilia cluster was identified in the fallopian tube in the sheep gene expression atlas (Clark et al., 2017) and a similar cluster was enriched in chicken in the trachea (Bush et al., 2018a). The goat mini-atlas also included several clusters that were enriched for immune tissues and cell types and we have based our analysis in part upon the premise that the greatest differences between small ruminant species likely involve the immune system.

### Gene Expression in the Neonatal Gastrointestinal Tract

Three regions of the gastrointestinal (GI) tract were sampled; the ileum, colon, and rumen. These regions formed distinct clusters in the network graph. The genes comprising these clusters were highly correlated with the physiology of the tissues. Goats are ruminant mammals and, at one-week of age (when tissues were collected), the rumen is vestigial. Even at this early stage of development, the typical epithelial signature of the rumen (Xiang et al., 2016a; Xiang et al., 2016b) was observed. Genes coexpressed in the rumen (clusters 7 and 13 – **Table 3**) were typical of a developing rumen epithelial signature (Bush et al., 2019) and were associated with GO terms for epidermis development (p = 0.00016), keratinocyte differentiation (p = 1.5x10^-14^), and skin morphogenesis (p = 8.2x10^-6^). Large colon (cluster 12) included several genes associated with GO terms for microvillus organization (p = 1x10^6^) and microvillus (p = 6.3x10^6^) including *MYO7B* which is found in the brush border cells of epithelial microvilli in the large intestine. The microvilli function as the primary surface of nutrient absorption in the gastrointestinal tract, and as such numerous phospholipid-transporting ATPases and solute carrier genes were found in the large colon cluster.

Throughout the GI tract, there was a strong immune signature, similar to that observed in neonatal and adult sheep (Bush et al., 2019), which was greatest in clusters 10 and 19 (**Table 3**) where expression was high in the ileum and Peyer’s patches, thymus, and spleen. Cluster 10 had a more general immune related profile with higher expression in the spleen and significant GO terms associated with cytokine receptor activity (p = 1.3x10^-8^) and T-cell receptor complex (p = 0.00895). Several genes involved in the immune and inflammatory response were found in cluster 10 including *CD74*, *IL10*, and *TLR10*. The expression pattern for cluster 19 was associated with B-cells including GO terms for B-cell proliferation (p = 1.4x10^-7^), positive regulation of B-cell activation (p = 4.9x10^-6^), and cytokine activity (p = 0.0051). Genes associated with the B-cell receptor complex *CD22*, *CD79B*, *CD180*, and *CR2*, and interleukins *IL21R* and *IL26* were expressed in cluster 19 (Treanor, 2012). This reflects the fact that we sampled the Peyer’s patch with the ileum, which is a primary lymphoid organ of B-cell development in ruminants (Masahiro et al., 2006).

Each of the GI tract clusters included genes associated with more than one cell type/cellular process. This complexity is a consequence of gene expression patterns from the lamina propria, one of the three layers of the mucosa. The lamina propria lies beneath the epithelium along the majority of the GI tract and comprises numerous different cell types from endothelial, immune and connective tissues (Ikemizu et al., 1994). This gene expression pattern, which is also observed in sheep (Clark et al., 2017; Bush et al., 2019) and pigs (Freeman et al., 2012), highlights the complex multidimensional physiology of the ruminant GI tract.

### Macrophage-Associated Signatures

A strong immune response is vitally important to neonatal mammals. Macrophages constitute a major component of the innate immune system acting as the first line of defense against invading pathogens and coordinating the immune response by triggering antimicrobial responses and other mediators of the inflammatory response (Hume, 2015). Several clusters in the goat mini-atlas exhibited a macrophage-associated signature. Cluster 11 (**Table 3**) contained several macrophage marker genes, including *CD68* which is expressed in AMs and BMDMs. The cluster includes the macrophage growth factor, *CSF1*, indicating that as in sheep (Clark et al., 2017), pigs (Freeman et al., 2012), and humans (Schroder et al., 2012) but in contrast to mice, according to the results of this study goat macrophages are autocrine for their own growth factor. GO terms associated with cluster 11 included phagocytosis (p = 3.5x10^-10^), inflammatory response (p = 1.4x10^-8^), and cytokine receptor activity (p = 0.00031). Many of the genes that were up-regulated in AMs in cluster 11, including C-type lectins *CLEC4A* and *CLEC5A*, have been shown to be down regulated in sheep (Clark et al., 2017; Bush et al., 2019), pigs (Freeman et al., 2012), and humans (Baillie et al., 2017) in the wall of the intestine. This highlights functional transcriptional differences in macrophage populations. AMs respond to microbial challenge as the first line of defense against inhaled pathogens. In contrast, macrophages in the intestinal mucosa down-regulate their response to microorganisms as a continuous inflammatory response to commensal microbes would be undesirable.

Cluster 11 (**Table 3**) also included numerous proinflammatory cytokines and chemokines which were up-regulated following challenge with lipopolysaccharide (LPS). Response to LPS was also reflected in several significant GO terms associated with this cluster including, cellular response to lipopolysaccharide (p = 5.8x10^-10^), and cellular response to cytokine stimulus (p = 9.5x10^-8^). C-type lectin *CLEC4E*, which is known to be involved in the inflammatory response (Baillie et al., 2017), interleukin genes such as *IL1B* and *IL27*, and *ADGRE1* were all highly inducible by LPS in BMDMs. *ADGRE1* (*EMR1,F4/80*) is a monocyte-macrophage marker involved in pattern recognition which exhibits interspecies variation both in expression level and response to LPS stimulation (Waddell et al., 2018). Based upon RNA-Seq data, ruminant genomes were found to encode a much larger form of *ADGRE1* than monogastric species, with complete duplication of the extracellular domain [44].

### Comparative Analysis of Macrophage-Associated Transcriptional Responses in Sheep and Goats

Transcriptional differences are linked to species-specific variation in response to disease, and have been widely documented in livestock (Bishop and Woolliams, 2014). For instance, ruminants differ in their response to a wide range of economically important pathogens. Variation in the expression of *NRAMP1* (*SLC11A1*) is involved in the response of sheep and goat to Johne’s disease (Cecchi et al., 2017). Similarly, resistance to *Haemonchus contortus* infections in sheep and goats is associated with a stronger Th2-type transcriptional immune response (Gill et al., 2000; Alba-Hurtado and Munoz-Guzman, 2013). To determine whether goats and sheep differ significantly in immune transcriptional signatures, we performed a comparative analysis of the macrophage samples from the goat mini-atlas and those included in our gene expression atlas for sheep (Clark et al., 2017). One caveat to this analysis that should be noted is that the sheep and goat samples were unfortunately not age-matched and as such differences in gene expression could be an effect of developmental stage rather than species-specific differences. However, as macrophage samples from both species were kept in culture prior to collection and analysis, we would expect the effect of developmental stage to be minimal.

**Figure 2:**
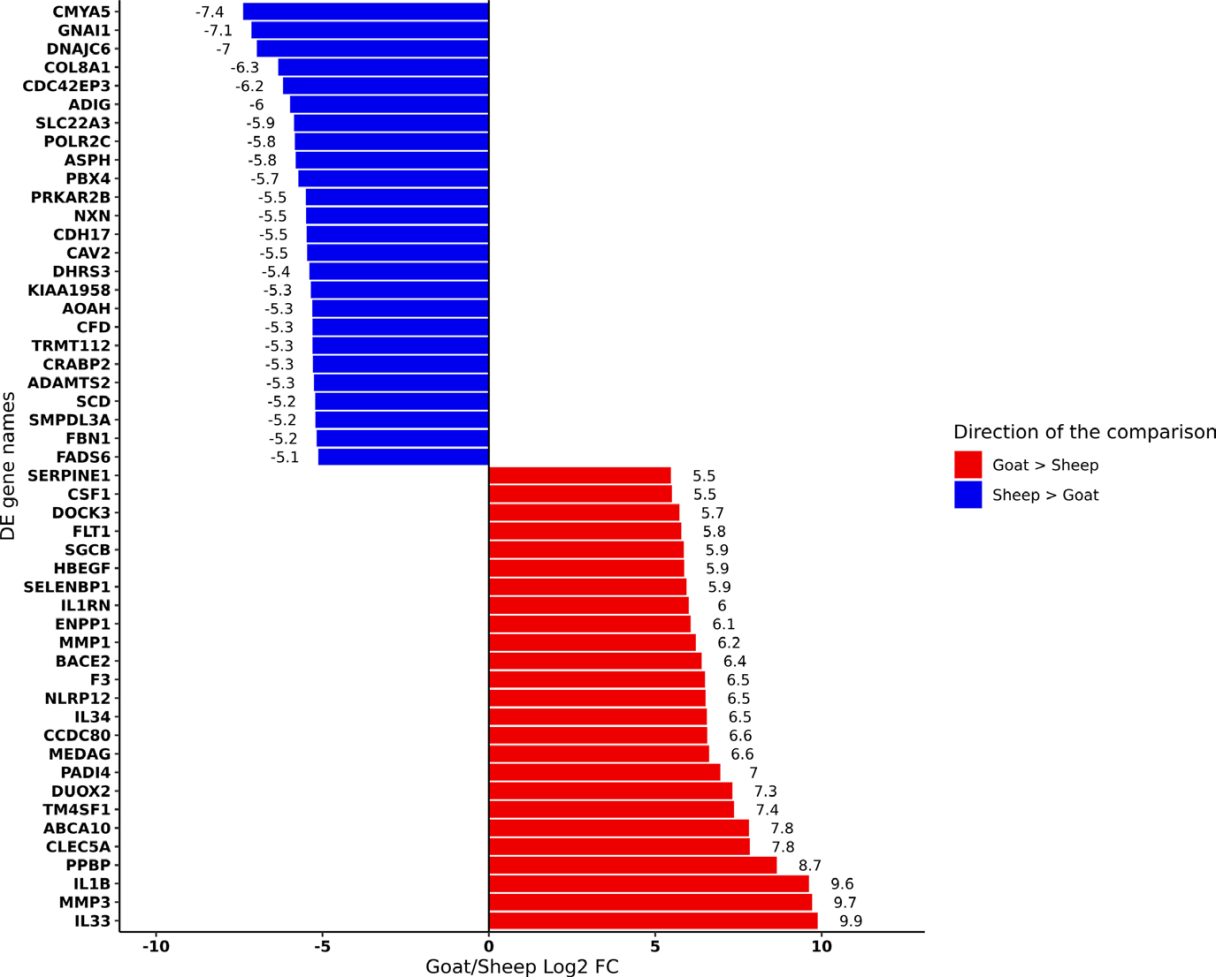
Differentially expressed genes (FDR < 10%) between goat and sheep alveolar macrophages. The top 25 up-regulated in goat relative to sheep (red) and the top 25 down-regulated in goat relative to sheep (blue) are shown.

We performed differential analysis of genes expressed in goat and sheep AMs (**Supplementary Table S13**). The top 25 genes up- and down-regulated in goat relative to sheep based on log2FC are shown in **Figure 2**. Several genes involved in the inflammatory and immune response including interleukins *IL33* and *IL1B* and C-type lectin *CLEC5A* were up-regulated in goat AMs relative to sheep. In contrast, those that were down regulated in goat relative to sheep did not have an immune function but were associated with more general physiological processes. This may reflect species-specific differences but could also indicate that the immune response in AMs is age-dependent, i.e., neonatal animals exhibit a primed immune response while a more subdued response is exhibited by adult sheep whose adaptive immunity has reached full development.

**Figure 3:**
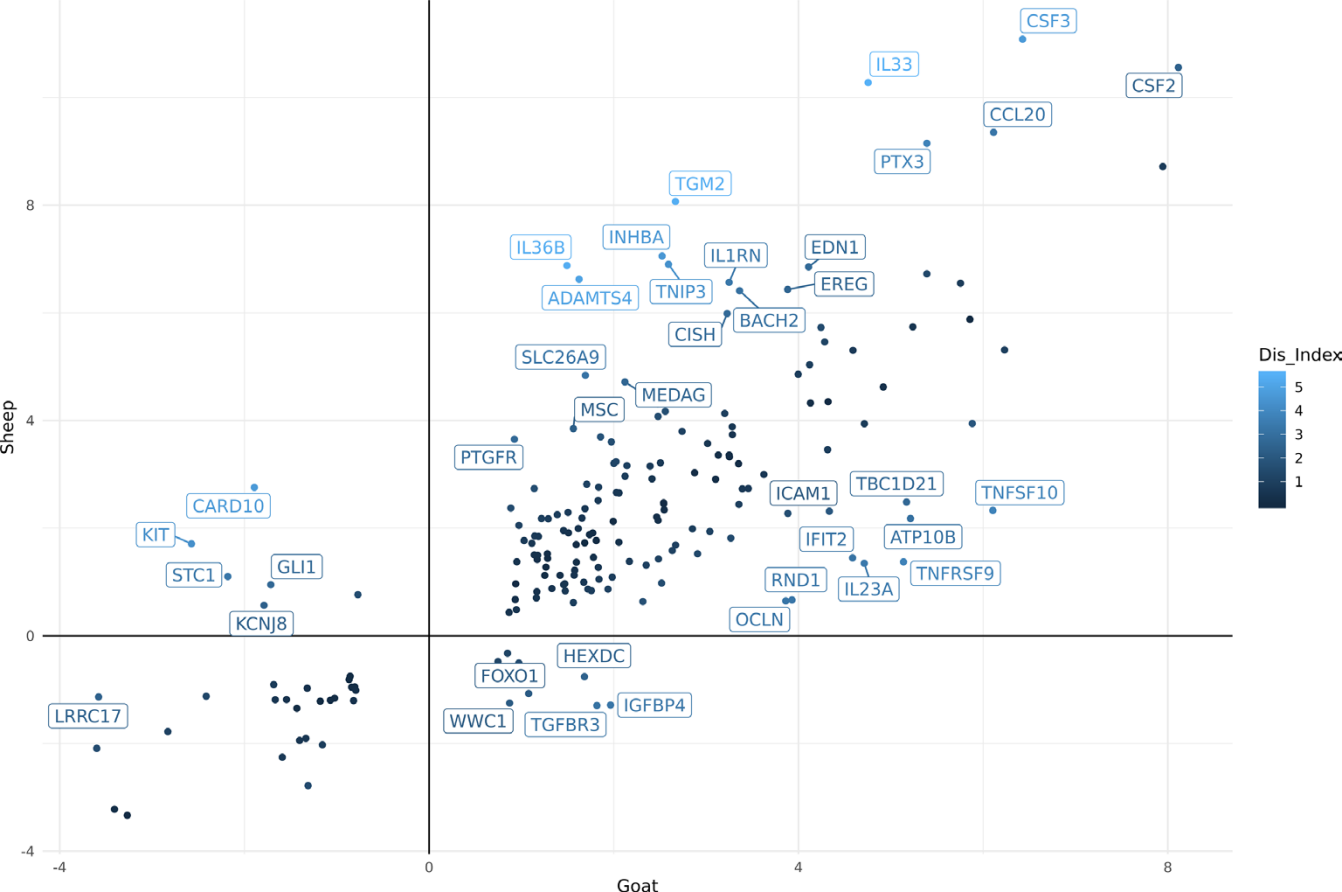
Comparative analysis of differentially expressed genes (FDR < 10%, Log2FC> = 2) in goat and sheep bone marrow derived macrophage (BMDM). The genes which showed the highest level of dissimilarity in response to lipopolysaccharide (LPS) between goats and sheep (Dis_Index> = 2) are shown. Top right quadrant: genes that were up-regulated in both goat and sheep but differed in their level of induction between the two species. Top left quadrant: genes that were up-regulated in sheep but down-regulated in goat. Bottom right quadrant: genes up-regulated in goat, but down-regulated in sheep.

Using differential expression analysis (Robinson et al., 2010), we also compared the gene expression estimates for sheep and goat BMDMs (+/-) LPS to compile a list of genes for each species that were up or down regulated in response to LPS (**Supplementary Table S14A** goat and **Supplementary Table S14B** sheep). These lists were then merged using the methodology described above (see Methods section) to highlight genes that differed in their response to LPS between the two species. In total, 188 genes exhibited significant differences between goats and sheep (FDR < 10%, Log2FC> = 2) in response to LPS (**Supplementary Table S15**). The genes which showed the highest level of dissimilarity in response to LPS between goats and sheep (Dis_Index> = 2) are illustrated in **Figure 3**. Several immune genes were upregulated in both goat and sheep BMDMs in response to LPS stimulation but differed in their level of induction between the two species (top right quadrant **Figure 3**). *IL33*, *IL36B*, *PTX3*, *CCL20, CSF3*, and *CSF2* for example, exhibited higher levels of induction in sheep BMDMs relative to goat, and vice versa for *ICAM1*, *IL23A*, *IFIT2*, *TNFSF10*, and *TNFRSF9*. Several genes were upregulated in sheep but downregulated in goat BMDMs (e.g., *KIT*) (top left quadrant **Figure 3**), and upregulated in goat, but downregulated in sheep (e.g., *IGFBP4*) (bottom right quadrant **Figure 3**).

Overall, the transcriptional patterns in BMDMs stimulated with LPS were broadly similar between the two species. Although, further experiments using qPCR to measure the expression of candidate genes in age-matched animals would be required to validate the observed expression patterns. With this caveat in mind, some interesting differences in individual genes were observed that could contribute to species-specific responses to infection. For instance, *IL33* and *IL23A* both exhibited a higher level of induction in sheep BMDMs after stimulation with LPS relative to goat (**Figure 3**). In humans, *IL33* has a protective role in inflammatory bowel disease by inducing a Th2 immune response (Lopetuso et al., 2013). An enhanced Th2 response, which accelerates parasite expulsion, has been associated with *H. contortus* resistance in sheep (Alba-Hurtado and Munoz-Guzman, 2013). Conversely, higher expression of *IL23A* is associated with susceptibility to *Teladorsagia circumcincta* infection (Gossner et al., 2012). Little is known about the function of *IL33* and *IL23A* in goats. They are members of the interleukin-1 family which play a central role in the regulation of immune and inflammatory response to infection (Dinarello, 2018). Given the similarities in their expression patterns, it is reasonable to assume that these genes are regulated in a similar manner to sheep and involved in similar biological pathways. As such, they would be suitable candidate genes to investigate further to determine if they underlie species-specific variation in susceptibility to pathogens (Bishop and Stear, 2003; Bishop and Morris, 2007).

### Expression Patterns of Genes Associated With Functional Traits in Goats

**Figure 4:**
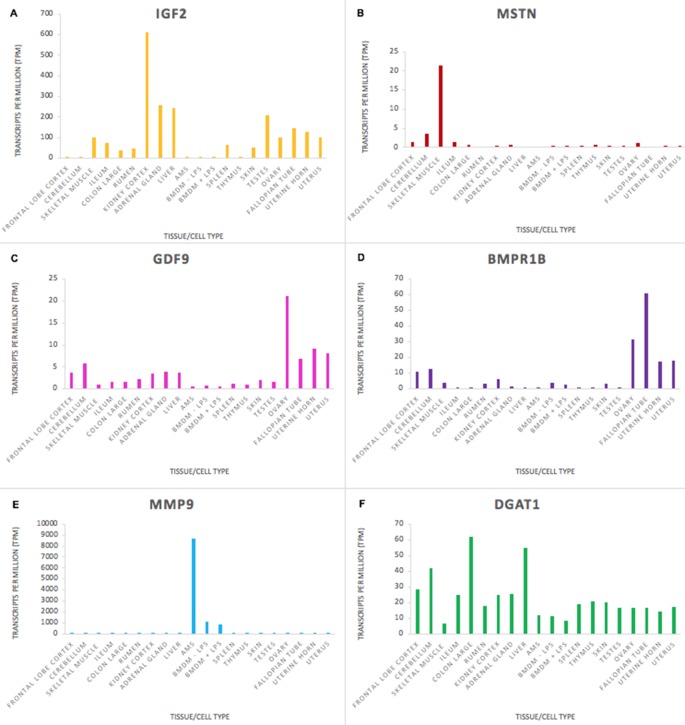
Expression levels (transcripts per million) of genes involved in functional traits in goats to illustrate tissue and cell type or ubiquitous expression patterns in the mini atlas dataset. **(A)** *IGF2* is associated with growth rate; **(B)** *MSTN* is associated with muscle characteristics; **(C)** *GDF9* is associated with ovulation rate; **(D)** *BMPR1* is associated with fecundity; **(E)** *MMP9 *is associated with resistance to mastitis; **(F)** *DGAT1* is associated with fat content in goat milk.

The goat mini-atlas dataset is a valuable resource that can be used by the livestock genomics community to examine the expression patterns of genes of interest that are relevant to ruminant physiology, immunity, welfare, production, and adaptation/resilience particularly in tropical agri-systems. The mini-atlas provides a resource of tissue-specific expression profiles for each gene that could be used to help determine which tissues to prioritise, for an expression QTL study, for example. Several genes, associated with functional traits in goats, have been identified using genome wide association studies (GWAS). Insulin-like growth factor 2 (*IGF2*), for example, is associated with growth rate in goats (Burren et al., 2016), and was highly expressed in tissues with a metabolic function including, kidney cortex, liver, and adrenal gland (**Figure 4A**). As expected, expression of myostatin (*MSTN*), which encodes a negative regulator of skeletal muscle mass, was highest in skeletal muscle in comparison with the other tissues (**Figure 4B**). *MSTN* is a target for gene-editing in goats to promote muscle growth (e.g., Yu et al., 2016). Expression of genes associated with fecundity and litter size in goats, including *GDF9* and *BMPR1B* (Feng et al., 2011; Shokrollahi and Morammazi, 2018), were highest in the ovary (**Figures 4C, D**). The ovary included here is from a neonatal goat and these results correlate with similar observations in sheep where genes essential for ovarian follicular growth and involved in ovulation rate regulation and fecundity were highly expressed in foetal ovary at 100 days gestation (Clark et al., 2017).

Some genes, particularly those involved in the immune response, had high tissue or cell type specific expression. Matrix metalloproteinase-9 (*MMP9*), which is involved in the inflammatory response and linked to mastitis regulation in goats (Li et al., 2016) was very highly expressed in macrophages, particularly AMs, in comparison with other tissues (**Figure 4E**). Other genes that are important for goat functional traits were fairly ubiquitously expressed. The expression level of Diacylglycerol O-Acyltransferase 1 (*DGAT1*) which is associated with milk fat content in dairy goats (Martin et al., 2017) did not vary hugely across the tissues sampled (**Figure 4F**), although there was slightly higher expression in some tissues (e.g., colon and liver) relative to immune tissues (e.g., thymus and spleen). *DGAT1* encodes a key metabolic enzyme that catalyses the last, and rate-limiting step of triglyceride synthesis, the transformation from a diacylglycerol to a triacylglycerol (Bell and Coleman, 1980). This is an important cellular process undertaken by the majority of cells, explaining its ubiquitous expression pattern. Two exonic mutations in the *DGAT1* gene in dairy goats have been associated with a notable decrease in milk fat content (Martin et al., 2017). Understanding how these, and other variants for functional traits, are expressed can help us to determine how their effect on gene expression and regulation influences the observed phenotypes in goat breeding programmes.

### Allele-Specific Expression

Using mapping bias correction for robust positive ASE discovery (Salavati et al., 2019), we were able to profile moderate to extreme allelic imbalance across tissues and cell types, at the gene level, in goats. The raw ASE values for every tissue/cell type are included in **Supplementary Dataset S4**. We first calculated the distribution of heterozygote sites per gene, as a measure of homogeneity of input sites, and found there was no significant difference between the eight individual goats included in the study (**Supplementary Figure S2**).

**Figure 5:**
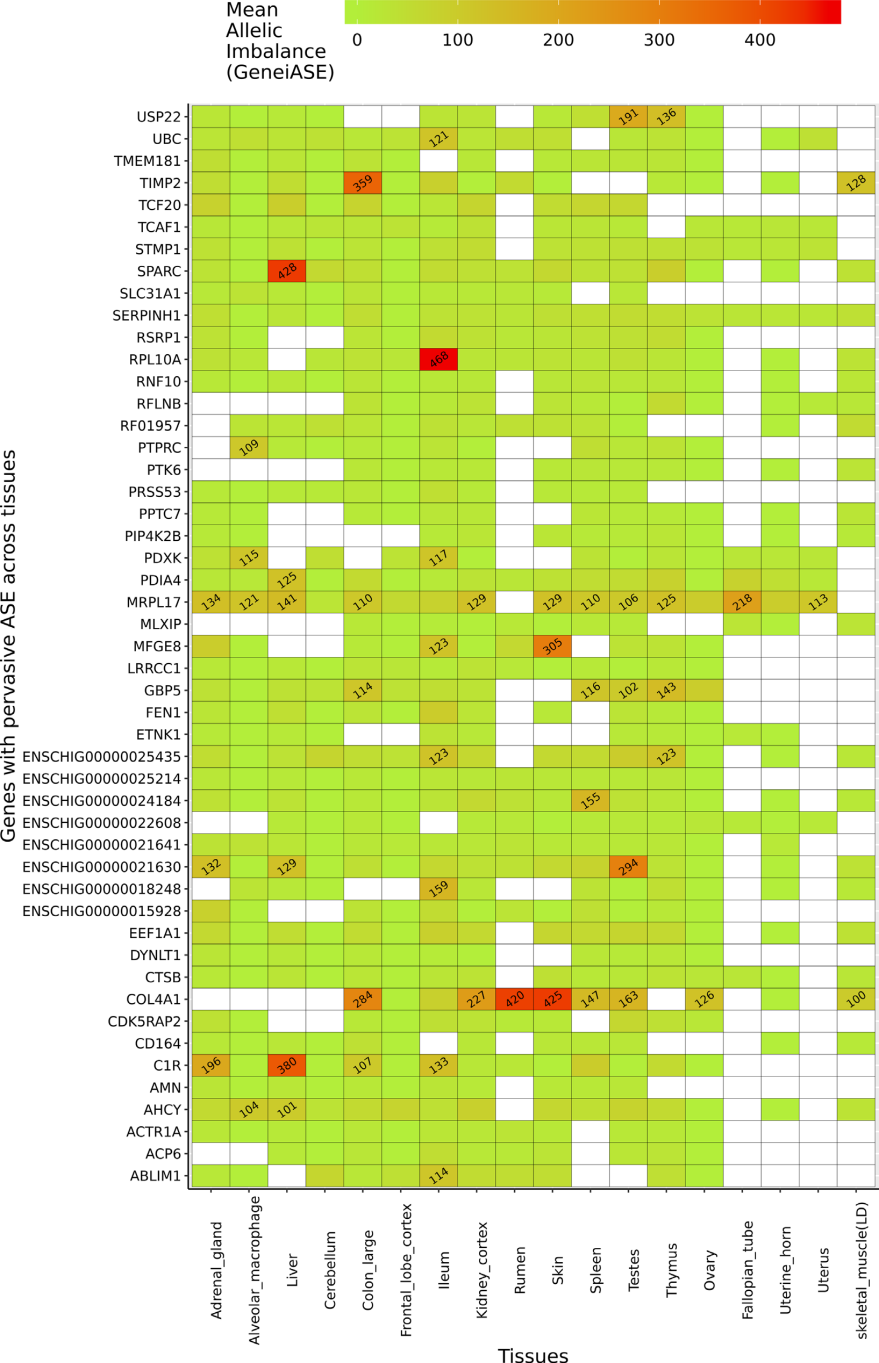
Genes exhibiting the largest mean allelic imbalance (i.e., allele-specific expression averaged across all heterozygote sites within each gene) across 17 tissues and one cell type from the goat mini-atlas dataset visualised as a heatmap (red indicating the highest level of mean allelic imbalance and green the least).

Several genes exhibited pervasive allelic imbalance (i.e., where the same imbalance in expression is shared across several tissues/cell types) (**Figure 5**). For example, allelic imbalance was observed in the mitochondrial ribosomal protein *MRPL17* in 16 tissues/cell types (except skeletal muscle and rumen). *SERPINH1*, a member of the serpin superfamily, was the only gene in which an imbalance in expression was detected in all tissues/cell types. Allelic imbalance was observed in *COL4A1* in 11 tissues, and was highest in the rumen and skin samples. *COL4A1* has been shown to be involved in the growth and development of the rumen papillae in cattle (Nishihara et al., 2018) and sheep (Bush et al., 2019). The highest levels of allelic imbalance in individual genes were observed in ribosomal protein *RPL10A* in ileum and *SPARC* in liver (**Figure 5**).

**Figure 6:**
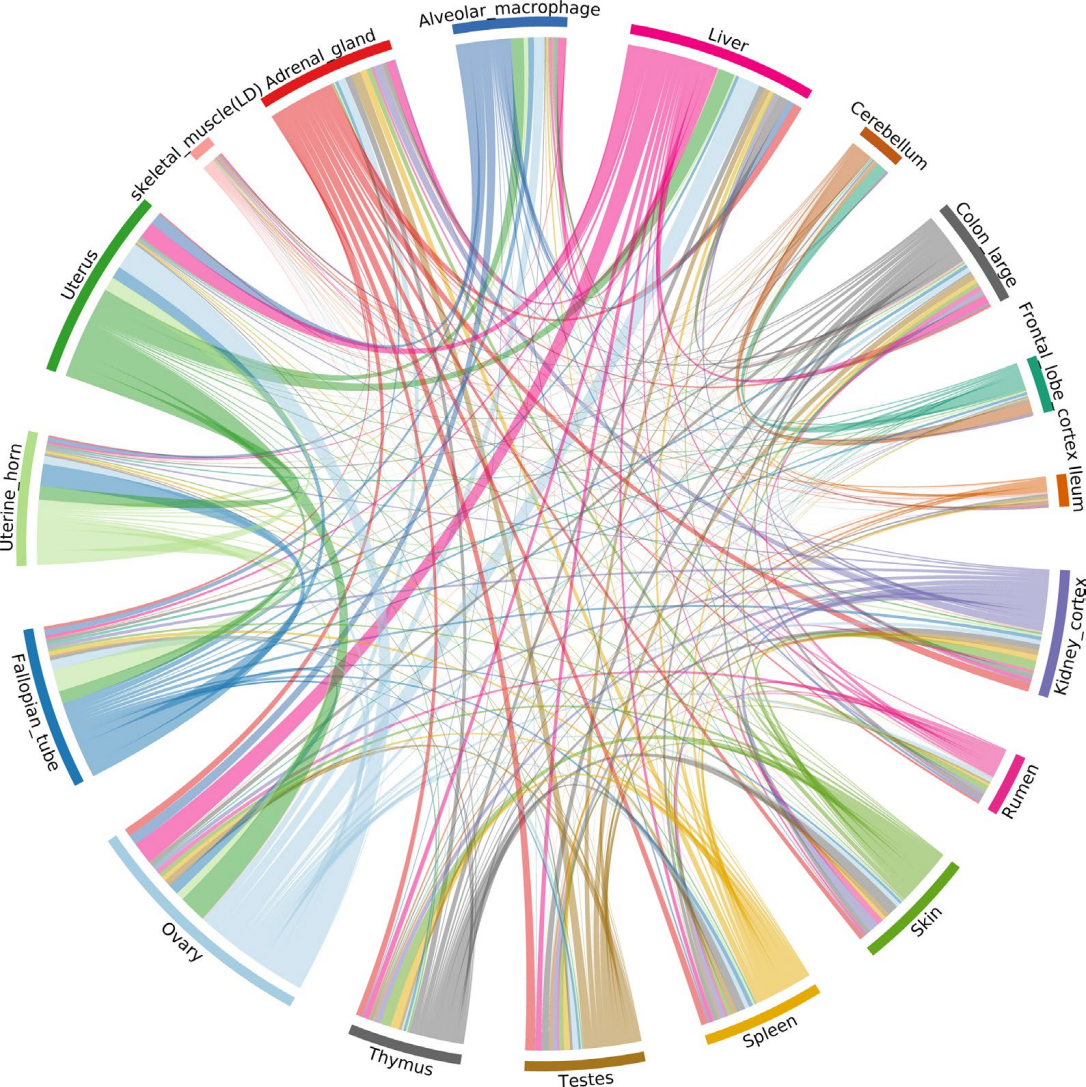
Correlation of allele-specific expression (ASE) profiles shared across tissues/cell types from the goat mini-atlas dataset. Each section represents the genes showing significant allelic imbalance within the tissue. The chords represent the correlation coefficient (CC < 0.85) of ASE profiles shared between the samples (i.e., the proportion of genes showing co-imbalance).

The ASE profiles were highly tissue- or cell type-specific, with strong correlations between samples from the same organ system (**Figure 6**). For example, ASE profiles in female reproductive system (ovary, fallopian tube, uterine horn, uterus), GI tract (colon and ileum), and brain (cerebellum and frontal lobe cortex) tissues were highly correlated. The two tissues showing the largest proportion of shared allele-specific expression were the ovary and liver (**Figure 6**). This might reflect transcriptional activity in these tissues in neonatal goats during oogenesis (ovary) and haematopoiesis (liver). Future work could determine if these ASE patterns were observed at other stages of development, or whether they are time-dependant.

The next step of this analysis would be to analyse ASE at the variant (SNV) level. This would allow us to identify variants driving ASE and determine whether they were located within important genes for functional traits. These variants could then be weighted in genomic prediction algorithms for genomic selection, for example. The sequencing depth used for the goat mini-atlas is, however, insufficient for statistically robust analysis at the SNV level. Nevertheless, it does provide a foundation for further analysis of ASE relevant to functional traits using a suitable dataset, ideally from a larger number of individuals (e.g., for aseQTL analysis (Wang et al., 2018)) and at a greater depth.

## Conclusions

We have created a mini-atlas of gene expression for the domestic goat. This expression dataset complements the genetic and genomic resources already available for goat (Tosser-Klopp et al., 2014; Stella et al., 2018; Talenti et al., 2018), and provides a set of functional information to annotate the current reference genome (Bickhart et al., 2017; Worley, 2017). We were able to detect the majority (90%) of the transcriptome from a subset of 17 transcriptionally rich tissues and 3 cell-types representing all the major organ systems, providing proof of concept that this mini-atlas approach is useful for studying gene expression and for functional annotation. Using the mini-atlas dataset, we annotated 15% of the unannotated genes in ARS1. Our dataset was also used by the Ensembl team to create a new gene build for the goat ARS1 reference genome (https://www.ensembl.org/Capra_hircus/Info/Index). One limitation of the mini-atlas is that it included only one biological replicate from a female goat because tissue from female dairy goats is difficult to source. Similarly, the samples used to generate the mini-atlas were all collected from neonatal animals and logistical constraints related to sample collection meant we could not sample immune cells from blood. Future studies could build on the mini-atlas, by including additional biological replicates from females, tissues from multiple developmental stages, and additional types of immune cell (e.g., monocytes, T-cells, and B-cells) to capture further transcriptional complexity.

We have also provided transcriptional profiling of macrophages in goats and a comparative analysis with sheep, which indicated in the cell types and animals investigated in this study transcriptional patterns in the two species were similar. This provides a foundation for further analysis in more tissues and cell types in age-matched animals, and in disease challenge experiments for example. Prior to this study, little was known about the transcription in goat macrophages. While more information is available on goat monocyte derived macrophages (Adeyemo et al., 1997; Taka et al., 2013; Walia et al., 2015), there was previously relatively little knowledge available on the characteristics of goat BMDMs. In addition, few reagents are available for immunological studies in goat, with most studies relying on cross-reactivity with sheep and cattle antibodies (Entrican, 2002; Hope et al., 2012). Recently, a characterisation of goat antibody loci has been published using the new reference genome ARS1 (Schwartz et al., 2018), demonstrating the usefulness of a highly contiguous reference genome with high quality functional annotation for the development of new resources for livestock species. The goat mini-gene expression atlas complements the large gene expression dataset we have generated for sheep and contributes to the genomic resources we are developing for interpretation of the relationship between genotype and phenotype in small ruminants.

## Data Availability Statement

We have made the files containing the expression estimates for the goat mini-atlas (**Supplementary Dataset S2** (unaveraged) and **Supplementary Dataset S3** (averaged)) available for download through the University of Edinburgh DataShare portal (https://doi.org/10.7488/ds/2591). Sample metadata for all the tissue and cell samples collected has been deposited in the EBI BioSamples database under project identifier GSB-2131 (https://www.ebi.ac.uk/biosamples/samples/SAMEG330351) according to FAANG metadata and data sharing standards. The raw fastq files for the RNA-Seq libraries are deposited in the European Nucleotide Archive (https://www.ebi.ac.uk/ena) under the accession number PRJEB23196. The data submission to the ENA includes experimental metadata prepared according to the FAANG Consortium metadata and data sharing standards. The BAM files are also available as analysis files under accession number PRJEB23196 (“BAM file 1” are mapped to the NCBI version of ARS1 and “BAM file 2” to the Ensembl version). The data from sheep included in this analysis has been published previously and is available *via* (Clark et al., 2017) and under ENA accession number PRJEB19199. Details of all the samples for both goat and sheep are available *via* the FAANG data portal (http://data.faang.org/home). All experimental protocols are available on the FAANG consortium website at http://www.ftp.faang.ebi.ac.uk/ftp/protocols. 

## Ethics Statement

The animal study was reviewed and approved by The Roslin Institute, University of Edinburgh’s Animal Work and Ethics Review Board (AWERB). All animal work was carried out under the regulations of the Animals (Scientific Procedures) Act 1986.

## Author Contributions

EC, CM, and DH designed the study. MA, AD, and DH provided guidance on project design, sample collection, and analysis. DH, MA, and AD secured the funding for the project with CM. CM and EC collected the samples with ZL and MM who performed the post mortems. CM performed the RNA extractions. SB performed the bioinformatic analyses. MS performed the analysis of allele-specific expression and assisted CM with the comparative analysis. CM performed the network cluster analysis with EC. CM and EC wrote the manuscript. All authors contributed to editing and approved the final version of the manuscript.

## Funding

This work was partially supported by a Biotechnology and Biological Sciences Research Council (BBSRC; www.bbsrc.ac.uk) grant BB/L001209/1 (‘Functional Annotation of the Sheep Genome’) and Institute Strategic Program grants ‘Blueprints for Healthy Animals’ (BB/P013732/1) and ‘Improving Animal Production and Welfare’ (BB/P013759/1). The goat RNA-seq data was funded by the Roslin Foundation (www.roslinfoundation.com), which also supported SB. CM was supported by a Newton Fund PhD studentship (www.newtonfund.ac.uk). EC is supported by a University of Edinburgh Chancellor’s Fellowship. Edinburgh Genomics is partly supported through core grants from the BBSRC (BB/J004243/1), National Research Council (NERC; www.nationalacademies.org.uk/nrc) (R8/H10/56), and Medical Research Council (MRC; www.mrc.ac.uk) (MR/K001744/1). Open access fees were covered by an RCUK block grant to the University of Edinburgh for article processing charges. The funders had no role in study design, data collection, and analysis, decision to publish, or preparation of the manuscript.

## Conflict of Interest

The authors declare that the research was conducted in the absence of any commercial or financial relationships that could be construed as a potential conflict of interest.

## References

[B1] Adeyemo O. Gao R. J. Lan H. C. (1997). Cytokine production in vitro by macrophages of goats with caprine arthritis-encephalitis. Cell Mol. Biol. 43 (7), 1031–1037. 9449535

[B2] Alba-Hurtado F. Munoz-Guzman M. A. (2013). Immune responses associated with resistance to haemonchosis in sheep. Biomed. Res. Int. 162158–162169. 10.1155/2013/162158 23509684 PMC3591228

[B3] Alexa A. Rahnenfuhrer J. (2010). topGO: Enrichment analysis for Gene Ontology [Online]. Available: http://www.bioconductor.org/packages/release/bioc/html/topGO.html.

[B4] Andersson L. Archibald A. L. Bottema C. D. Brauning R. Burgess S. C. Burt D. W. (2015). Coordinated international action to accelerate genome-to-phenome with FAANG, the Functional Annotation of Animal Genomes project. Genome Biol 16 (1), 57. 10.1186/s13059-015-0622-4 25854118 PMC4373242

[B5] Baillie J. K. Arner E. Daub C. De Hoon M. Itoh M. Kawaji H. (2017). Analysis of the human monocyte-derived macrophage transcriptome and response to lipopolysaccharide provides new insights into genetic aetiology of inflammatory bowel disease. PLoS Genet 13 (3), e1006641. 10.1371/journal.pgen.1006641 28263993 PMC5358891

[B6] Bell R. M. Coleman R. A. (1980). Enzymes of Glycerolipid Synthesis in Eukaryotes. Annu. Rev. Biochem. 49 (1), 459–487. 10.1146/annurev.bi.49.070180.002331 6250446

[B7] Bickhart D. M. Rosen B. D. Koren S. Sayre B. L. Hastie A. R. Chan S. (2017). Single-molecule sequencing and chromatin conformation capture enable *de novo* reference assembly of the domestic goat genome. Nat. Genet. 49 (4), 643–650. 10.1038/ng.3802 28263316 PMC5909822

[B8] Bishop S. C. Morris C. A. (2007). Genetics of disease resistance in sheep and goats. Small Rumin Res 70 (1), 48–59. 10.1016/j.smallrumres.2007.01.006

[B9] Bishop S. C. Stear M. J. (2003). Modeling of host genetics and resistance to infectious diseases: understanding and controlling nematode infections. Vet Parasitol 115 (2), 147–166. 10.1016/s0304-4017(03)00204-8 12878420

[B10] Bishop S. C. Woolliams J. A. (2014). Genomics and disease resistance studies in livestock. Livest Sci 166, 190–198. 10.1016/j.livsci.2014.04.034 26339300 PMC4547482

[B11] Bray N. L. Pimentel H. Melsted P. Pachter L. (2016). Near-optimal probabilistic RNA-seq quantification. Nat. Biotech. 34, 525–527. 10.1038/nbt.3519 27043002

[B12] Burren A. Neuditschko M. Signer-Hasler H. Frischknecht M. Reber I. Menzi F. (2016). Genetic diversity analyses reveal first insights into breed-specific selection signatures within Swiss goat breeds. Anim. Genet. 47 (6), 727–739. 10.1111/age.12476 27436146

[B13] Bush S. J. Freem L. MacCallum A. J. O’Dell J. Wu C. Afrasiabi C. (2018a). Combination of novel and public RNA-seq datasets to generate an mRNA expression atlas for the domestic chicken. BMC Genomics 19 (1), 594–594. 10.1186/s12864-018-4972-7 30086717 PMC6081845

[B14] Bush S. J. McCulloch M. E. B. Muriuki C. Salavati M. Davis G. M. Farquhar I. L. (2019). Comprehensive transcriptional profiling of the gastrointestinal tract of ruminants from birth to adulthood reveals strong developmental stage specific gene expression. G3 (Bethesda) 9 (2), 359. 10.1534/g3.118.200810 30530642 PMC6385975

[B15] Bush S. J. Muriuki C. McCulloch M. E. B. Farquhar I. L. Clark E. L. Hume D. A. (2018b). Cross-species inference of long non-coding RNAs greatly expands the ruminant transcriptome. GSE 50 (1), 20. 10.1186/s12711-018-0391-0 29690875 PMC5926538

[B16] Cecchi F. Russo C. Iamartino D. Galiero A. Turchi B. Fratini F. (2017). Identification of candidate genes for paratuberculosis resistance in the native Italian Garfagnina goat breed. Trop Anim Health Prod 49 (6), 1135–1142. 10.1007/s11250-017-1306-8 28526988

[B17] Clark E. L. Bush S. J. McCulloch M. E. B. Farquhar I. L. Young R. Lefevre L. (2017). A high resolution atlas of gene expression in the domestic sheep (Ovis aries). PLOS Genet 13 (9), e1006997. 10.1371/journal.pgen.1006997 28915238 PMC5626511

[B18] Dinarello C. A. (2018). Overview of the IL-1 family in innate inflammation and acquired immunity. Immunol Rev. 281 (1), 8–27. 10.1111/imr.12621 29247995 PMC5756628

[B19] Dong Y. Xie M. Jiang Y. Xiao N. Du X. Zhang W. (2013). Sequencing and automated whole-genome optical mapping of the genome of a domestic goat (Capra hircus). Nat. Biotechnol. 31 (2), 135–141. 10.1038/nbt.2478 23263233

[B20] Edsgärd D. Iglesias M. J. Reilly S.-J. Hamsten A. Tornvall P. Odeberg J. (2016). GeneiASE: Detection of condition-dependent and static allele-specific expression from RNA-seq data without haplotype information. Sci Rep 6, 21134. 10.1038/srep21134 26887787 PMC4758070

[B21] Entrican G. (2002). New technologies for studying immune regulation in ruminants. Vet Immunol and Immunopathol 87, (3–4), 485–490. 12072276 10.1016/s0165-2427(02)00082-x

[B22] FAANG (2017). FAANG Data Portal [Online]. Available: http://data.faang.org/home.

[B23] Feng T. Geng C. X. Lang X. Z. Chu M. X. Cao G. L. Di R. (2011). Polymorphisms of caprine GDF9 gene and their association with litter size in Jining Grey goats. Mol Biol Rep 38 (8), 5189–5197. 10.1007/s11033-010-0669-y 21181498

[B24] Freeman T. C. Goldovsky L. Brosch M. van Dongen S. Mazière P. (2007). Construction, visualisation and clustering of transcription networks from micorarray expression data. PLoS Comp. Biol. 3 (10), e206. 10.1371/journal.pcbi.0030206 PMC2041979 17967053

[B25] Freeman T. C. Ivens A. Baillie J. K. Beraldi D. Barnett M. W. Dorward D. (2012). A gene expression atlas of the domestic pig. BMC Biol. 10 (1), 90. 10.1186/1741-7007-10-90 23153189 PMC3814290

[B26] Gill H. S. Altmann K. Cross M. L. Husband A. J. (2000). Induction of T helper 1- and T helper 2-type immune responses during Haemonchus contortus infection in sheep. Immunol. 99 (3), 458–463. 10.1046/j.1365-2567.2000.00974.x PMC2327170 10712677

[B27] Gossner A. G. Venturina V. M. Peers A. Watkins C. A. Hopkins J. (2012). Expression of sheep interleukin 23 (IL23A, alpha subunit p19) in two distinct gastrointestinal diseases. Vet Immunol Immunopathol 150 (1–2), 118–122. 10.1016/j.vetimm.2012.08.004 22939273

[B28] Harrison P. W. Fan J. Richardson D. Clarke L. Zerbino D. Cochrane G. (2018). FAANG, establishing metadata standards, validation and best practices for the farmed and companion animal community. Anim. Genet. 49 (6), 520–526. 10.1111/age.12736 30311252 PMC6334167

[B29] Hope J. C. Sopp P. Wattegedera S. Entrican G. (2012). Tools and reagents for caprine immunology. Small Rumin Res 103 (1), 23–27. 10.1016/j.smallrumres.2011.10.015

[B30] Hume D. A. (2015). The Many Alternative Faces of Macrophage Activation. Front. Immunol. 6, 370–370. 10.3389/fimmu.2015.00370 26257737 PMC4510422

[B31] Ikemizu T. Kitamura N. Yamada J. Yamashita T. (1994). Is Lamina Muscularis Mucosae Present in the Ruminal Mucosa of Cattle? Anat. Histol. Embryol. 23 (2), 177–186. 10.1111/j.1439-0264.1994.tb00250.x 7978352

[B32] Kim D. Langmead B. Salzberg S. L. (2015). HISAT: a fast spliced aligner with low memory requirements. Nat. Meth. 12 (4), 357–360. 10.1038/nmeth.3317 PMC4655817 25751142

[B33] Krupp M. Marquardt J. U. Sahin U. Galle P. R. Castle J. Teufel A. (2012). RNA-Seq Atlas - A reference database for gene expression profiling in normal tissue by next generation sequencing. Bioinf. 28 (8), 1184–1185. 10.1093/bioinformatics/bts084 22345621

[B34] Larroque H. Rupp R. Conington J. Mucha S. McEwan J. (2016). Genomic application in sheep and goat breeding. Anim. Front. 6 (1), 39–44. 10.2527/af.2016-0006

[B35] Li H. Zheng H. Li L. Shen X. Zang W. Sun Y. (2016). The Effects of Matrix Metalloproteinase-9 on Dairy Goat Mastitis and Cell Survival of Goat Mammary Epithelial Cells. PLoS One 11 (8), e0160989. 10.1371/journal.pone.0160989 27518717 PMC4982621

[B36] Livigni A. O’Hara L. Polak M. E. Angus T. Wright D. W. Smith L. B. (2018). A graphical and computational modeling platform for biological pathways. Nat. Protoc. 13, 705. 10.1038/nprot.2017.144 29543794

[B37] Lopetuso L. R. Chowdhry S. Pizarro T. T. (2013). Opposing Functions of Classic and Novel IL-1 Family Members in Gut Health and Disease. Front. Immunol. 4, 181. 10.3389/fimmu.2013.00181 23847622 PMC3705591

[B38] Martin P. Palhière I. Maroteau C. Bardou P. Canale-Tabet K. Sarry J. (2017). A genome scan for milk production traits in dairy goats reveals two new mutations in Dgat1 reducing milk fat content. Sci Rep 7 (1), 1872. 10.1038/s41598-017-02052-0 28500343 PMC5431851

[B39] Masahiro Y. Craig N. J. Laurie J. K. John D. R. (2006). The sheep and cattle Peyer’s patch as a site of B-cell development. Vet Res 37 (3), 401–415. 16611555 10.1051/vetres:2006008

[B40] Muriuki C. Bush S. J. Salavati M. McCulloch M. E. B. Lisowski Z. M. Agaba M. (2019). A mini-atlas of gene expression for the domestic goat (*Capra hircus*) reveals transcriptional differences in immune signatures between sheep and goats. BioRxiv. 10.1101/711127 PMC6844187 31749840

[B41] Nishihara K. Kato D. Suzuki Y. Kim D. Nakano M. Yajima Y. (2018). Comparative transcriptome analysis of rumen papillae in suckling and weaned Japanese Black calves using RNA sequencing. J. Anim. Sci. 96 (6), 2226–2237. 10.1093/jas/skx016 29762736 PMC6095377

[B42] Oliver S. (2000). Proteomics: Guilt-by-association goes global. Nat. 403 (6770), 601–603. 10.1038/35001165 10688178

[B43] Pertea M. Pertea G. M. Antonescu C. M. Chang T.-C. Mendell J. T. Salzberg S. L. (2015). StringTie enables improved reconstruction of a transcriptome from RNA-seq reads. Nat. Biotech. 33 (3), 290–295. 10.1038/nbt.3122 PMC4643835 25690850

[B44] Pruitt K. D. Tatusova T. Maglott D. R. (2007). NCBI reference sequences (RefSeq): a curated non-redundant sequence database of genomes, transcripts and proteins. Nucleic Acids Res. 35, D61–D65. 10.1093/nar/gkl842 17130148 PMC1716718

[B45] Pulina G. Milán M. J. Lavín M. P. Theodoridis A. Morin E. Capote J. (2018). Invited review: Current production trends, farm structures, and economics of the dairy sheep and goat sectors. J. Dairy. Sci. 101 (8), 6715–6729. 10.3168/jds.2017-14015 29859690

[B46] Robinson M. D. McCarthy D. J. Smyth G. K. (2010). edgeR: a Bioconductor package for differential expression analysis of digital gene expression data. Bioinf. 26 (1), 139–140. 10.1093/bioinformatics/btp616 PMC2796818 19910308

[B47] Salavati M. Bush S. J. Palma-Vera S. McCulloch M. E. B. Hume D. A. Clark E. L. (2019). Elimination of reference mapping bias reveals robust immune related allele-specific expression in cross-bred sheep. Front. Genet. 10:863. 10.3389/fgene.2019.00863 31608110 PMC6761296

[B48] Schroder K. Irvine K. M. Taylor M. S. Bokil N. J. Le Cao K.-A. Masterman K.-A. (2012). Conservation and divergence in Toll-like receptor 4-regulated gene expression in primary human versus mouse macrophages. PNAS 109 (16), E944–E953. 10.1073/pnas.1110156109 22451944 PMC3341041

[B49] Schwartz J. C. Philp R. L. Bickhart D. M. Smith T. P. L. Hammond J. A. (2018). The antibody loci of the domestic goat (Capra hircus). Immunogenet. 70 (5), 317–326. 10.1007/s00251-017-1033-3 PMC5899754 29063126

[B50] Sherman D. M. (2011). The spread of pathogens through trade in small ruminants and their products. Rev Sci Tech, 30 (1), 207–217. 21809765 10.20506/rst.30.1.2036

[B51] Shokrollahi B. Morammazi S. (2018). Polymorphism of GDF9 and BMPR1B genes and their association with litter size in Markhoz goats. Repro Domest Anim 53 (4), 971–978. 10.1111/rda.13196 29696699

[B52] Stella A. Nicolazzi E. L. Van Tassell C. P. Rothschild M. F. Colli L. Rosen B. D. (2018). AdaptMap: exploring goat diversity and adaptation. GSE 50 (1), 61. 10.1186/s12711-018-0427-5 30453882 PMC6240945

[B53] Taka S. Liandris E. Gazouli M. Sotirakoglou K. Theodoropoulos G. Bountouri M. (2013). In vitro expression of the SLC11A1 gene in goat monocyte-derived macrophages challenged with Mycobacterium avium subsp paratuberculosis. Infect. Genet. Evol. 17, 8–15. 10.1016/j.meegid.2013.03.033 23567820

[B54] Talenti A. Palhière I. Tortereau F. Pagnacco G. Stella A. Nicolazzi E. L. (2018). Functional SNP panel for parentage assessment and assignment in worldwide goat breeds. GSE 50 (1), 55. 10.1186/s12711-018-0423-9 30449282 PMC6240953

[B55] Tosser-Klopp G. Bardou P. Bouchez O. Cabau C. Crooijmans R. Dong Y. (2014). Design and Characterization of a 52K SNP Chip for Goats. PLoS One 9 (1), e86227. 10.1371/journal.pone.0086227 24465974 PMC3899236

[B56] Treanor B. (2012). B-cell receptor: from resting state to activate. Immunol. 136 (1), 21–27. 10.1111/j.1365-2567.2012.03564.x PMC3372753 22269039

[B57] van de Geijn B. McVicker G. Gilad Y. Pritchard J. K. (2015). WASP: allele-specific software for robust molecular quantitative trait locus discovery. Nat. Meth. 12, 1061. 10.1038/nmeth.3582 PMC4626402 26366987

[B58] Waddell L. A. Lefevre L. Bush S. J. Raper A. Young R. Lisowski Z. M. (2018). ADGRE1 (EMR1, F4/80) Is a Rapidly-Evolving Gene Expressed in Mammalian Monocyte-Macrophages. Front. Immunol. 9, 2246. 30327653 10.3389/fimmu.2018.02246 PMC6174849

[B59] Walia V. Kumar R. Mitra A. (2015). Lipopolysaccharide and Concanavalin A Differentially Induce the Expression of Immune Response Genes in Caprine Monocyte Derived Macrophages. Anim. Biotechnol. 26 (4), 298–303. 10.1080/10495398.2015.1013112 26158463

[B60] Wang M. Hancock T. P. Chamberlain A. J. Vander Jagt C. J. Pryce J. E. Cocks B. G. (2018). Putative bovine topological association domains and CTCF binding motifs can reduce the search space for causative regulatory variants of complex traits. BMC Genomics 19, 395. 10.1186/s12864-018-4800-0 29793448 PMC5968476

[B61] Wang M. Yu H. Kim Y. S. Bidwell C. A. Kuang S. (2012). Myostatin facilitates slow and inhibits fast myosin heavy chain expression during myogenic differentiation. Biochem. Biophys. Res. Commun. 426 (1), 83–88. 10.1016/j.bbrc.2012.08.040 22910409 PMC3483024

[B62] Wickham H. François R. Henry L. Müller K., (2018). dplyr: A Grammar of Data Manipulation. R package version 0.7.6. [Online]. Available: https://CRAN.R-project.org/package=dplyr.

[B63] Wickramasinghe S. Cánovas A. Rincón G. Medrano J. F. (2014). RNA-Sequencing: A tool to explore new frontiers in animal genetics. Livestock Sci. 166, 206–216. 1016/j.livsci.201406.015

[B64] Worley K. C. (2017). A golden goat genome. Nat. Genet. 49 (4), 485–486. 10.1038/ng.3824 28358125

[B65] Xiang R. McNally J. Rowe S. Jonker A. Pinares-Patino C. S. Oddy V H. (2016a). Gene network analysis identifies rumen epithelial cell proliferation, differentiation and metabolic pathways perturbed by diet and correlated with methane production. Sci Rep 6, 39022. 10.1038/srep39022 27966600 PMC5155297

[B66] Xiang R. Oddy V. H. Archibald A. L. Vercoe P. E. Dalrymple B. P. (2016b). Epithelial, metabolic and innate immunity transcriptomic signatures differentiating the rumen from other sheep and mammalian gastrointestinal tract tissues. PeerJ 4, e1762. 10.7717/peerj.1762 26989612 PMC4793311

[B67] Young R. Bush S. J. Lefevre L. McCulloch M. E. B. Lisowski Z. M. Muriuki C. (2018). Species-Specific Transcriptional Regulation of Genes Involved in Nitric Oxide Production and Arginine Metabolism in Macrophages. ImmunoHorizons 2 (1), 27. 30467554 10.4049/immunohorizons.1700073 PMC6245571

[B68] Yu B. Lu R. Yuan Y. Zhang T. Song S. Qi Z. (2016). Efficient TALEN-mediated myostatin gene editing in goats. BMC Dev. Biol. 16 (1), 26. 10.1186/s12861-016-0126-9 27461387 PMC4962387

[B69] Zerbino D. R. Achuthan P. Akanni W. AmodeBarrell M. R.D. Bhai J. (2018). Ensembl 2018. Nucleic Acids Res. 46 (D1), D754–D761. 10.1093/nar/gkx1098 29155950 PMC5753206

