# Supplementary figures and images for: A Mini-Atlas of Gene Expression for the Domestic Goat (Capra hircus)
Source: Front Genet. 2019 Nov 4;10:1080. doi: 10.3389/fgene.2019.01080 (PMC6844187; doi:10.3389/fgene.2019.01080)

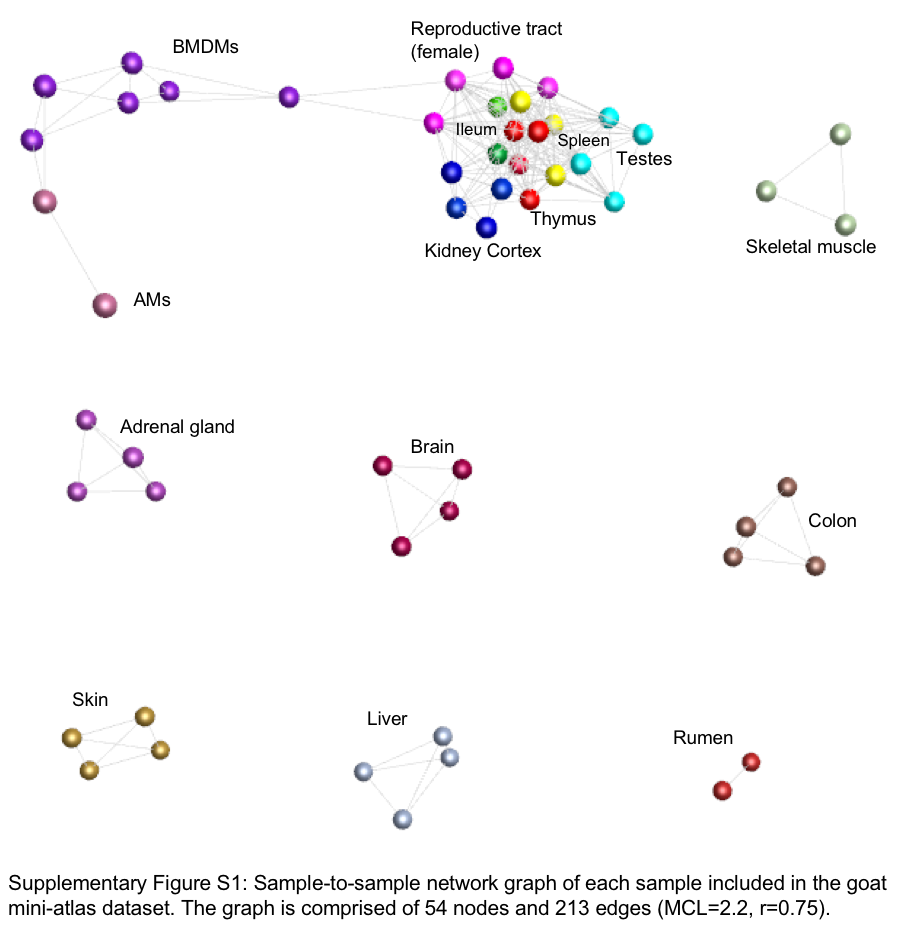

Supplement: Supplementary file 5 [file Image_1.tiff]

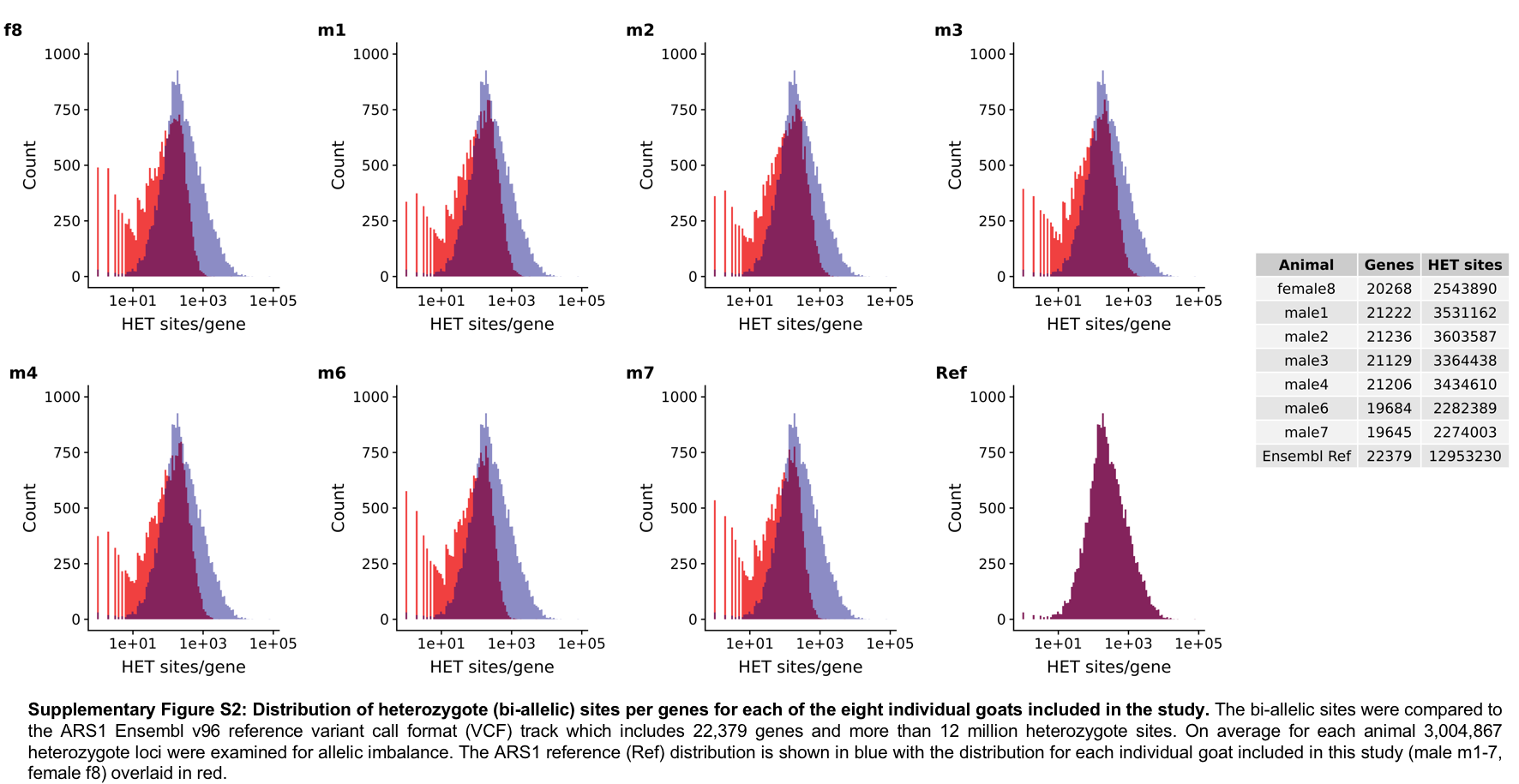

Supplement: Supplementary file 6 [file Image_2.tiff]
